# The Effect of Temperature and Humidity on Yellow Tea Volatile Compounds during Yellowing Process

**DOI:** 10.3390/foods13203283

**Published:** 2024-10-16

**Authors:** Weiwei Wang, Zhihui Feng, Rui Min, Junfeng Yin, Heyuan Jiang

**Affiliations:** Key Laboratory of Tea Biology and Resources Utilization, Ministry of Agriculture, Tea Research Institute, Chinese Academy of Agricultural Sciences, Hangzhou 310008, China; wangwei11211@tricaas.com (W.W.); fengzhihui@tricaas.com (Z.F.); minrui@tricaas.com (R.M.); yinjf@tricaas.com (J.Y.)

**Keywords:** yellow tea, volatile compounds, temperature, humidity

## Abstract

Yellowing is the key processing technology of yellow tea, and environmental conditions have a significant impact on the yellowing process. In this study, volatile compounds of the yellowing process under different environmental conditions were analyzed by GC–MS. Results showed that a total of 75 volatile compounds were identified. A partial least squares discriminant analysis (PLS-DA) determined that 42 of them were differential compounds, including 12 hydrocarbons, 8 ketones, 8 aldehydes, 6 alcohols, and 8 other compounds, and compared the contents of differential compounds under the conditions of 40 °C with 90% humidity, 50 °C with 50% humidity, and 30 °C with 70% humidity, then analyzed the variation patterns of hydrocarbons under different yellowing environmental conditions. A 40 °C with 90% humidity treatment reduced the content of more hydrocarbons and increased the aldehydes. The content of 3-hexen-1-ol was higher when treated at 50 °C with 50% humidity and was consistent with the results of sensory evaluation. This study could provide a theoretical basis for future research on the aroma of yellow tea.

## 1. Introduction

Yellow tea, which has the characteristics of “yellow dry tea, yellow tea infusion, and yellow brewed tea leaves”, is one of the six traditional teas of China. Yellow tea is a lightly fermented tea with antioxidant [1], anti-inflammatory, and anticancer effects which has a history of more than 1200 years [2,3]. The main producing areas of Chinese yellow tea include Sichuan, Hunan, Anhui, Hubei, Zhejiang, and other provinces. Yellow tea is divided into bud yellow tea, little yellow tea, and large-leaf yellow tea. Pingyang yellow tea is one type of little yellow tea produced in Pingyang County, Zhejiang province, with a history of over 200 years. In 2022, the output of Pingyang yellow tea was 1.85 million tons, with a value of CNY 280 million. The processing technology of Pingyang yellow tea mainly includes withering, fixation, rolling, yellowing and drying, among which yellowing is the key process of yellow tea [4]. Under the influence of hither, the color of tea changes from green to yellow gradually during the yellowing process, and the fresh and sweet components such as amino acids and sucrose increase, accompanied by chlorophyll degradation, flavan-3-ols decomposition, the reduction of catechins, flavonol glycosides, and caffeine leads to a decrease in bitterness [5,6,7]. The temperature and humidity during the yellowing process are particularly important for the quality of yellow tea, and they are the key factors for forming the yellowness and sweetness of yellow tea color. Studies have shown that increasing the yellowing temperature from 20 °C to 34 °C and the relative humidity from 55% to 67% concurrently could increase the yellowness and sweetness of yellow tea and improv the quality of yellow tea [8].

Aroma, composed of numerous volatile compounds with low content and significant differences, is one of the important factors of tea quality. Volatile compounds occurred in about 0.01% of dry tea weight [9]. Yellow tea has five aroma types such as chestnut, floral, corn, high-fire, and fresh. The flavor of yellow tea is cleaner than green tea [10]. Aromatic compounds form in the hydrolysis reaction of amino acids and glycosides during yellow tea processing, wherein the sweet, floral, and corn-like aromas are mainly affected by yellowing [11]. In addition, the Maillard reaction occurs to form volatile compounds with sweet and floral flavors during the drying process of yellow tea. The studies found that the proportion of alcohols and aldehydes increased during the yellowing process of Pingyang yellow tea, the content of geraniol, linalool, phenylacetaldehyde, linalool oxide, and cis-3-hexenol increased with the prolongation of yellowing time [12]. There is still little research on the aroma of yellow tea. In this study, learning from the processing technology of Pingyang yellow tea, samples of yellow tea using different yellowing temperatures and humidities were collected, and we analyzed the influence of environmental conditions on the volatile compounds during the yellowing process. This study provides a theoretical basis for optimizing the technical parameters of yellow tea.

## 2. Materials and Methods

### 2.1. Tea Leaves

For yellow tea processing, the tea trees of the Longjing 43 cultivar were selected as the source of fresh tea leaves. The tea leaves with one bud and two leaves were plucked for the processing experiments in September 2023 from the tea garden in Shengzhou City, Zhejiang, China.

### 2.2. Yellow Tea Processing and Samplings

**Figure 1:**
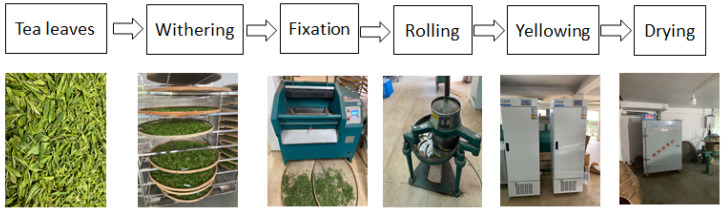
Flowchart of yellow tea process.

1. Withering: the fresh tea leaves (5 kg) were spread on a bamboo mat (thickness was 1–3 cm) for 16 h at room temperature until the moisture level reached 70–72%.
2. Fixation: the withering tea leaves were subjected to a fixation machine (6CCB-45 type)
3. Rolling: the fixation tea leaves were rolled for 30 min in a rolling machine (6CR-25 type).
4. Yellowing: The rolling tea leaves were divided into three equal portions, then we put two of them into an artificial climate box, which was purchased from Ningbo prandt instrument Co., Ltd., Ningbo, China, with the yellowing environment temperatures and humidities of 40 °C with 90% simultaneously (H) and 50 °C with 50% simultaneously (L), using natural treatment (30 °C with 70% simultaneously) as control (D).
5. Samplings: A total of 100 g of each treatment of yellowing for 2 h, 4 h, 6 h, and 8 h were dried at 120 °C and collected as the tea samples.
6. Yellow tea processing flowchart: The fresh leaves, processing equipment were shown in Figure 1.

### 2.3. Preparation of Tea Broth Solution and Internal Standard

A total of 1 g tea samples were weighed out and placed in 50 mL boiling water, covered and soaked in a 100 °C water bath for 4 min. The tea soup was filtered through 400-mesh gauze, then cooled down to room temperature quickly using an ice bath for subsequent experiments. Take 10 mL tea soup to a headspace flask, then add 5 ug ethyl caprate (the concentration was 8.64 ppm), and mix thoroughly through vortexing. Headspace solid-phase microextraction (SPME, from Sigma-Aldrich, St. Louis, MO, USA) was performed in a water bath at 30 °C, followed by adsorption for 45 min.

### 2.4. Gas Chromatography–Mass Spectrometry (GC–MS) Analysis

The volatile concentrates of yellow tea samples were analyzed using a GC–MS system (Agilent Technologies 7890B gas chromatograph equipped with a mass selective detector of 5977B, Agilent, Santa Clara, CA, USA) and performed by DB-WAX capillary column (30 m × 0.32 mm, 0.25 μm) with a linear velocity of 30 cm/sec carrier gas (helium). GC oven temperature was programmed as follows: at an initial temperature of 40 °C for 5 min, followed by a temperature increase to 100 °C at 3 °C/min, then to 200 °C at 5 °C/min, and finally to 250 °C at 15 °C/min, then maintained for 7 min. The mass spectrometry was performed using electron ionization (EI) mode with a scan range of 30 to 350 *m*/*z* at 70 eV. The ion source temperature was 230 °C, and the quadrupole temperature was 150 °C. Volatile compounds were identified by retention indices (RIs), authentic standards and mass spectra matching in the standard NIST14 library. An n-alkane mixture (C 6–C 40) was used for the determination of RIs.

### 2.5. Sensory Evaluation

Sensory evaluation was completed by eight panelists (three men and two women) from the Tea Research Institute. They were trained in professional sensory evaluation of tea before the sensory experiments using the tea brewing and evaluation reference GB/T 23776-2018, “Methodology for sensory evaluation of tea”. A total of 3 g tea samples were weighed out and placed in 150 mL boiling water for 5 min. The intensities were scored (0–10, 0 means non-perceptible, 10 means strongly perceived), and aroma attributes were agreed among the panelists.

### 2.6. Statistical Analysis

All the data were preprocessed by the mean. The principal component analysis (PCA), partial least squares discriminant analysis (PLS-DA), was used to investigate the tea samples using SMICA 14.0 software for multivariate analysis. The heat map was drawn by multiple experiment viewer software.

## 3. Results and Discussion

### 3.1. Sensory Evaluation of Yellow Teas

**Figure 2:**
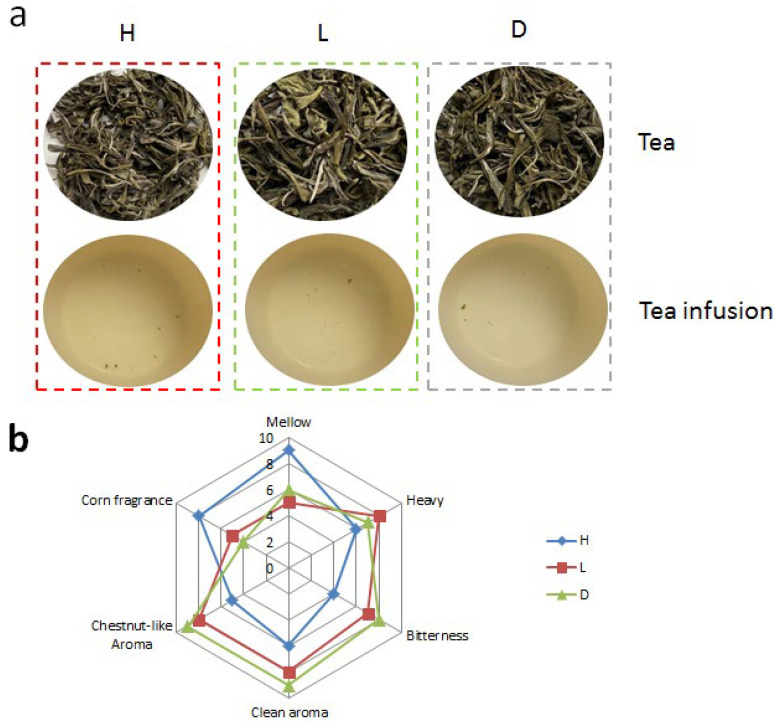
(**a**) Tea and tea infusion. (**b**) Radar map of the sensory of yellow tea.

Quantitative description analysis of sensory attributes was conducted on three types of yellow teas. There are significant differences in the appearance color and tea infusion color of yellow tea treated with different temperature and humidity treatments (Figure 2a). The two colors of 40 °C with 90% humidity treatment were more yellow and had the more distinct characteristics of yellow tea quality. It focused on two components: aroma and flavor (Figure 2b). In particular, aroma was classified into three categories: clean aroma, chestnut-like aroma, and corn fragrance. The flavor was classified into three categories: mellow, heavy, and bitterness. Among all samples, 40 °C with 90% humidity treatment had the highest intensities of mellow and corn fragrance, and it had the lowest intensities of clean aroma, chestnut-like aroma, and bitterness. The 40 °C with 90% humidity treatment had a significant difference compared with the other two treatments.

### 3.2. Analysis of Volatile Compounds

Yellow tea has a unique aroma quality due to the starch hydrolysis and protein decomposition during the special yellowing process [13]. Studies showed that alcohols, esters, aldehydes, ketones, hydrocarbons, and alkenes were found to be abundant volatiles in yellow tea [11]. In this study, the volatile compounds of yellow tea samples and their processing samples were determined by GC–MS. The results showed that a total of 75 volatile compounds were identified (Table 1), involving 17 hydrocarbons, 15 aldehydes, 14 ketones, 11 alcohols, 5 oxygen heterocyclic compounds, 3 esters, and 10 other compounds. In addition to the main alcohols, aldehydes, and ketones, this study also analyzed a large number of alkanes, and alkanes were also the most abundant compounds found in Longjing tea [14].

Among these volatile compounds, 2-Methyl-butanal and 3-Methyl-butanal were found to be the most abundant in yellow tea, 2-Methyl-butanal might be partially generated from amino acids that were derived from protein hydrolysis in the yellowing process [15]. 2-Ethyl-1-hexanol and 1-Pentanol were the highest content among alcohols; 4-Methyl-3-penten-2-one was the highest content among ketones; 2,4-Dimethyl-1-heptene was the highest content among hydrocarbons. As an important aroma component in tea, linalool was found to have a higher content under 40 °C with 90% humidity (0.51 μg/Kg) and 50 °C with 50% humidity (0.48 μg/Kg) treatment compared with natural yellowing (0.24 μg/kg). In addition, previous studies on yellow tea had also found 1,3-dimethyl-benzene, which was considered a bad aroma compound [16]. This study found that the 40 °C with 90% humidity treatment had a lower content of 1,3-Dimethyl-benzene compared with natural yellowing.

### 3.3. Differential Volatile Compounds Analysis of Different Treatments Yellow Tea

**Figure 3:**
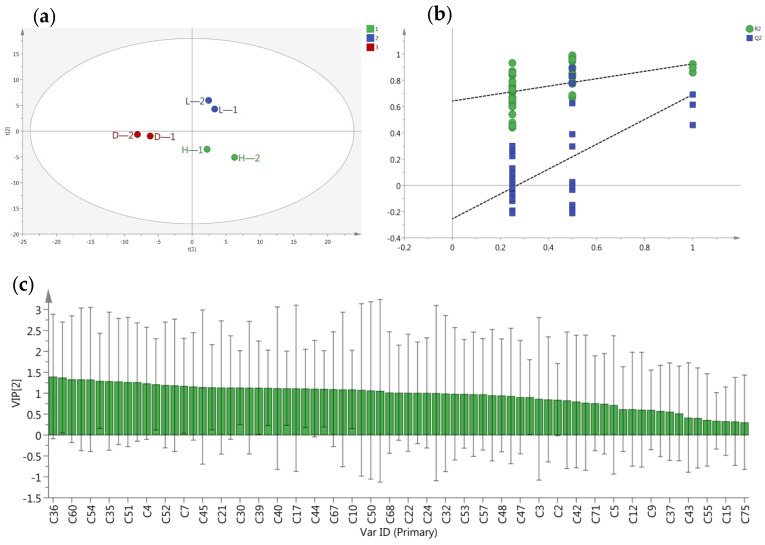
Metabolomics analysis of volatile compounds in different yellow teas: PLS-DA score plot (**a**), cross-validation plot (**b**), VIP plot (**c**).

The partial least squares discriminant analysis found that different temperature and humidity treatments were distributed clearly on the score plot (Figure 3a); the 40 °C with 90% humidity and 50 °C with 50% humidity treatments were in quadrants 1 and 4 and located on the right of the *y*-axis, and the 30 °C with 70% humidity treatment was in quadrant 3. As shown in Figure 3c, 42 volatile compounds for the classification of tea were 1-penten-3-ol, furan, decanal, 2,3-pentanedione, 6-ethyl-2-methyl-decane, 2,2,6-trimethyl-cyclohexanone, 1,3-dimethyl-benzene, 6,10-dimethyl-,(Z) -5,9-undecadien-2-one, nonanal, etc. (VIP > 1 and *p* < 0.05) (Figure 3c) Among the differential metabolites, hydrocarbon compounds have the most types, accounting for 12, followed by aldehydes and ketones with 8 types, alcohols with 6 types, and a remaining 8 other compounds. The cross-validation results showed no over-fitting of the model with 200 iterations (intercepts, R^2^ = 0.664 and Q^2^ = −0.214) (Figure 3b).

### 3.4. The Influence of Temperature and Humidity on Yellow Tea

**Figure 4:**
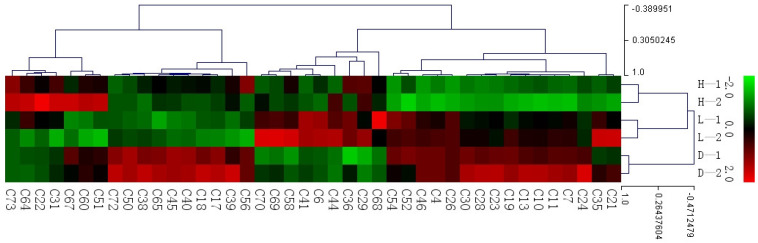
Heat map of differential volatile compounds in yellow teas.

To show the aroma compounds of yellow tea samples with different treatments more clearly, a heat map of 42 differential compounds is constructed (Figure 4), in which red represents high contents and blue represents low contents. 2,4-dimethyl-decane, 2,4-dimethyl-heptane, 4-methyl-octane, 3-ethyl-hexane, 2,4-dimethyl-1-heptene, 3,3-dimethyl-2-butanone, 4-methyl-decane, 3,7-dimethyl-nonane, 5-ethyl-2-methyl-octane, 2,3-pentanedione, 2,3-dimethyl-hexane, and 2,2,6-trimethyl-cyclohexanone showed the lowest contents in the 40 °C with 90% humidity treatment, and the contents in the 50 °C with 50% humidity treatment was also lower than the control (30 °C with 70% humidity). 4-methyl-1-hexanol, 3-methyl-1-butanol, 2-Methyl-butanal, 3-methyl-butanal, 4-methyl-undecane, 4,7-dimethyl-undecane, 2-methyl-propanoic acid, and acetyl valeryl showed the lowest contents in the 50 °C with 50% humidity treatment, and the contents in the 40 °C with 90% humidity treatment were also lower than the control (30 °C with 70% humidity). Among them, 3-methyl-1-butanol had a bitter, sweet, and heavily astringent taste and a watery, dull odor, which was considered a bad aroma compound in yellow tea [17]. The contents of octanal, furan, 2-propenyl ester butanoic acid, 1-decen-3-one, 2-amino-5-methyl-phenol, and 2,6,6-trimethyl-1-cyclohexene-1-carboxaldehyde in the 50 °C with 50% humidity treatment were higher than that in the control (30 °C with 70% humidity). The contents of nonanal, decanal, undecanal, hexanal, pentanal, 1-octanol, and 6,10-dimethyl-,(Z)-5,9-undecadien-2-one in the 40 °C with 90% humidity treatment were higher than that in the control (30 °C with 70% humidity). It can be seen that the 40 °C with 90% humidity treatment has a significant effect on the aroma components of yellow tea. It reduced the content of nine different hydrocarbons in a total of 11 compounds and increased the content of five different aldehydes in a total of 8 compounds. The high-temperature treatment reduced the content of some components that were unfavorable to the quality of yellow tea.

3-Methylbutanal, 3-hexen-1-ol, 1-octen-3-ol, octanal, phenylacetaldehyde, 1-octanol, linalool, dimethyl sulfide, geraniol, β-damascenone, jasmone, and β-ionone were considered to have a significant contribution to the sweet and floral corn-like aroma [18]. Benzaldehyde had an almond aroma and increased significantly during the yellowing process [19]. In this study, benzaldehyde, 3-methylbutanal, 3-hexen-1-ol, 1-octen-3-ol, octanal, phenylacetaldehyde, 1-octanol, and linalool were identified, and we found that seven compounds except 3-methylbutanal had a higher content under 40 °C with 90% humidity and 50 °C with 50% humidity treatment. Among them, benzaldehyde, 3-hexen-1-ol, 1-octen-3-ol, phenylacetaldehyde, 1-octanol, and linalool were higher under 40 °C with 90% humidity treatment; thus, it can be seen that high humidity treatment is beneficial for the formation of the aroma quality of yellow tea.

### 3.5. Changes in the Differential Compounds of Hydrocarbons during the Yellowing Process

**Figure 5:**
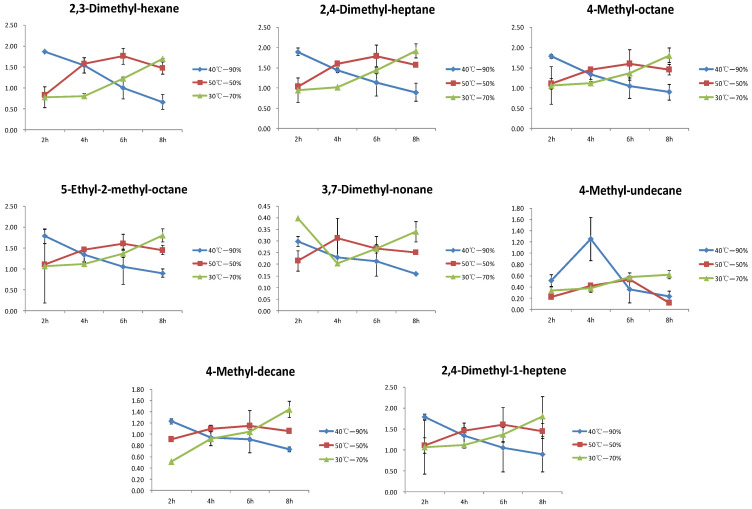
Trend chart of changes in alkanes during the yellowing process.

This study identified a significant number of hydrocarbons, and also found that hydrocarbons accounted for the largest proportion among the differential compounds through PLS-DA analysis. This paper analyzed the variation patterns of eight hydrocarbons under different temperature and humidity conditions (Figure 5). The variation pattern of 2,3-Dimethyl-hexane, 2,4-Dimethyl-heptane, 4-Methyl-octane, 5-Ethyl-2-methyl-octane, 4-Methyl-decane, and 2,4-Dimethyl-1-heptene in different treatments were consistent, all showing gradual decreasing during the yellowing process at 40 °C with 90% humidity treatment, gradual increasing during the yellowing process at 30 °C with 70% humidity treatment, and an initial increase followed by a decrease during the yellowing process at 50 °C with 50% humidity treatment. After 8 h of yellowing treatment, the contents of six compounds at 30 °C with 70% humidity treatment were the highest, followed by the 50 °C with 50% humidity treatment, and the 40 °C with 90% humidity treatment had the lowest contents. 3,7-Dimethyl-nonane of the 30 °C with 70% humidity treatment showed a pattern of an initial decrease followed by an increase, and the 40 °C with 90% humidity and 50 °C with 50% humidity treatments were consistent with the previous six compounds. 4-Methyl-undecane of the 30 °C with 70% humidity treatment showed a gradually increasing trend, and the 40 °C with 90% humidity and 50 °C with 50% humidity treatments showed a trend of an initial increase, then decrease.

## 4. Conclusions

The temperature and humidity environmental conditions had a significant impact on the yellowing process of yellow tea. Using different temperatures and humidities to treat the yellowing process of yellow tea, this study set three different conditions: high temperature and low humidity (50 °C, 50%), high humidity (40 °C, 90%), and natural control (30 °C, 70%). Yellow tea and the processing samples were analyzed by GC–MS, and 75 volatile compounds were identified. Through the PLS-DA analysis, it was found that the three treatments can be well distinguished on the score graph. A total of 42 differential compounds were screened based on VIP > 1 and *p* < 0.05 as the standard. The 40 °C with 90% humidity treatment reduced the content of the nine hydrocarbons in eleven differential hydrocarbons, increased benzaldehyde, 3-hexen-1-ol, 1-octen-3-ol, octanal, phenylacetaldehyde, 1-octanol, linalool contents, and increased the content of the five aldehydes in eight differential aldehydes. Decanal was considered to be associated with floral and fruity aromas, and 3-hexen-1-ol was considered to be associated with fresh and grassy aromas [10]. This study found that the content of decanal was higher when treated at 40 °C with 90% humidity, and the content of 3-hexen-1-ol was higher when treated at 50 °C with 50% humidity. It was consistent with the results of the sensory evaluation. Further analysis of the changes in hydrocarbons during the yellowing process found that most differential hydrocarbons showed a gradual decrease during the yellowing process at 40 °C with 90% humidity treatment but a gradual increase during the yellowing process at 30 °C with 70% humidity treatment. Overall, this study provides a theoretical basis for future research on the aroma of yellow tea.

## Figures and Tables

**Table 1:** Volatile compounds of yellow tea identified by GC–MS.

No.	RT	CAS	RI	Volatile Compounds	Mean Content (μg/kg)	Content Range
C1	4.260	591-93-5	686	1,4-Pentadiene	1.00	0.78–1.28
C2	4.435	74-93-1	706	Methanethiol	0.25	0.21–0.29
C3	4.584	109-92-2	713	Ethoxyethene	0.47	0.40–0.61
C4	5.042	584-94-1	740	2,3-Dimethyl-hexane	1.28	0.66–1.70
C5	5.492	926-56-7	813	4-Methyl-1,3-pentadiene	1.60	1.08–1.88
C6	5.549	110-00-9	816	Furan	0.77	0.35–1.57
C7	5.610	2213-23-2	819	2,4-Dimethyl-heptane	1.46	0.89–1.92
C8	5.762	78-84-2	827	2-Methyl propanal	6.42	5.30–7.82
C9	6.055	79-20-9	840	Acetic acid, methyl ester	2.62	2.42–2.89
C10	6.378	619-99-8	855	3-Ethyl-hexane	0.19	0.19–0.24
C11	6.484	2216-34-4	859	4-Methyl-octane	1.38	0.90–1.80
C12	6.900	534-22-5	877	2-Methyl-furan	0.86	0.78–0.91
C13	7.036	19549-87-2	883	2,4-Dimethyl-1-heptene	3.26	2.33–4.12
C14	7.284	141-78-6	893	Ethyl acetate	1.32	1.09–1.68
C15	7.505	534-22-5	901	2-Methyl-furan	0.46	0.44–0.48
C16	7.608	78-93-3	905	2-Butanone	1.22	1.13–1.29
C17	7.950	96-17-3	918	2-Methyl-butanal	58.73	50.93–70.27
C18	8.091	590-86-3	923	3-Methyl-butanal	19.80	17.68–22.82
C19	9.137	75-97-8	959	3,3-Dimethyl-2-butanone	0.19	0.12–0.25
C20	9.272	3208-16-0	963	2-Ethyl-furan	0.76	0.70–0.86
C21	10.075	3710-43-8	988	2,4-Dimethylfuran	0.35	0.28–0.41
C22	10.162	110-62-3	990	Pentanal	9.65	8.20–12.05
C23	10.582	2847-72-5	1002	4-Methyl-decane	1.07	0.73–1.44
C24	12.409	2801-84-5	1049	2,4-Dimethyl-decane	0.36	0.21–0.57
C25	12.684	4170-30-3	1055	2-Butenal	0.16	0.14–0.17
C26	13.577	600-14-6	1075	2,3-Pentanedione	0.16	0.00–0.48
C27	13.732	25044-01-3	1079	2-Methyl-1-penten-3-one	0.79	0.73–0.89
C28	13.952	17302-32-8	1083	3,7-Dimethyl-nonane	0.25	0.16–0.34
C29	14.052	624-92-0	1086	Dimethyl-disulfide	0.42	0.29–0.48
C30	14.177	62016-18-6	1088	5-Ethyl-2-methyl-octane	1.46	0.93–2.01
C31	14.406	66-25-1	1093	Hexanal	3.11	2.72–4.33
C32	14.798	3744-02-3	1101	4-Methyl-4-penten-2-one	1.51	1.25–1.91
C33	16.578	79-46-9	1137	2-Nitro-propane	1.58	1.31–1.72
C34	16.798	141-79-7	1141	4-Methyl-3-penten-2-one	11.05	10.40–12.26
C35	17.026	108-38-3	1145	1,3-Dimethyl-benzene	1.42	0.99–1.88
C36	18.166	616-25-1	1167	1-Penten-3-ol	0.54	0.00–0.88
C37	19.230	617-92-5	1188	1-Ethyl-1-H-pyrrole	2.95	2.56–3.53
C38	19.554	96-04-8	1194	Acetyl valeryl	0.29	0.13–0.58
C39	20.338	123-51-3	1209	3-Methyl-1-butanol	1.04	0.36–1.70
C40	20.589	2980-69-0	1214	4-Methyl-undecane	0.32	0.11–0.62
C41	22.172	2051-78-7	1246	2-Propenyl ester butanoic acid	0.09	0.00–0.17
C42	22.342	71-41-0	1249	1-Pentanol	1.45	1.29–1.70
C43	22.783	100-42-5	1259	Styrene	1.13	1.07–1.18
C44	24.195	124-13-0	1289	Octanal	0.86	0.38–1.40
C45	24.306	17301-32-5	1291	4,7-Dimethyl-undecane	0.68	0.24–1.28
C46	25.404	2408-37-9	1316	2,2,6-Trimethyl-cyclohexanone	0.38	0.00–0.85
C47	25.875	585-25-1	1327	2,3-Octanedione	0.66	0.35–0.88
C48	26.417	110-93-0	1340	6-Methyl-5-hepten-2-one	2.14	0.92–3.01
C49	26.953	111-27-3	1352	1-Hexanol	0.21	0.19–0.24
C50	28.260	544-12-7	1385	3-Hexen-1-ol	0.14	0.00–0.22
C51	28.626	124-19-6	1394	Nonanal	1.66	0.97–2.22
C52	29.373	15726-15-5	1414	3-Methyl-4-heptanone	0.15	0.09–0.19
C53	29.821	1014-60-4	1426	1,3-Bis(1,1-dimethylethyl)-benzene	1.18	0.99–1.44
C54	30.175	62108-21-8	1435	6-Ethyl-2-methyl-decane	0.24	0.11–0.32
C55	30.579	996-12-3	1446	2,2-Dimethyl-hexanal	0.47	0.43–0.53
C56	30.732	818-49-5	1451	4-Methyl-1-hexanol	0.16	0.09–0.22
C57	31.368	98-01-1	1469	Furfural	0.37	0.30–0.44
C58	31.752	56606-79-2	1479	1-Decen-3-one	0.27	0.20–0.36
C59	31.872	104-76-7	1483	2-Ethyl-1-hexanol	4.00	2.75–4.89
C60	32.256	112-31-2	1494	Decanal	3.85	0.99–5.99
C61	33.014	109-97-7	1516	Pyrrole	0.36	0.34–0.37
C62	33.260	100-52-7	1524	Benzaldehyde	1.19	0.96–1.40
C63	33.649	78-70-6	1536	Linalool	0.41	0.24–0.48
C64	33.918	111-87-5	1544	1-Octanol	0.20	0.16–0.26
C65	34.428	79-31-2	1560	2-Methyl-propanoic acid	0.40	0.28–0.54
C66	34.704	620-02-0	1568	5-Methyl-2-furancarboxaldehyde	0.12	0.12–0.13
C67	35.307	112-44-7	1588	Undecanal	0.20	0.09–0.26
C68	35.460	29957-43-5	1593	3,7-Dimethyl-1,5,7-octatrien-3-ol	1.34	0.58–2.25
C69	35.727	2835-98-5	1601	2-Amino-5-methyl-phenol	0.28	0.22–0.38
C70	35.937	432-25-7	1608	2,6,6-Trimethyl-1-cyclohexene-1-carboxaldehyde	0.12	0.10–0.16
C71	36.664	122-78-1	1632	Benzeneacetaldehyde	1.31	0.89–1.63
C72	37.141	116-53-0	1648	2-Methyl-butanoic acid	0.15	0.09–0.27
C73	41.460	3879-26-3	1806	6,10-Dimethyl-,(Z)-5,9-undecadien-2-one	5.14	1.00–12.20
C74	41.800	120-50-3	1820	2-Methylpropyl ester benzoic acid	0.19	0.16–0.23
C75	41.877	74367-30-9	1823	2-Methyl-, 2-ethyl-1-propyl-1,3-propanediyl ester-propanoic acid	11.76	11.05–12.49

## Data Availability

The original contributions presented in the study are included in the article. Further inquiries can be directed to the corresponding author.
